# Anaesthesia in austere environments: literature review and considerations for future space exploration missions

**DOI:** 10.1038/s41526-018-0039-y

**Published:** 2018-02-23

**Authors:** Matthieu Komorowski, Sarah Fleming, Mala Mawkin, Jochen Hinkelbein

**Affiliations:** 1 Department of Surgery and Cancer, Faculty of Medicine, Imperial College London, Exhibition road, London, SW7 2AZ UK; 2 Space Medicine Team, ISS Operations and Astronauts Group, European Astronaut Centre, European Space Agency, Linder Hoehe, Köln, 51147 Germany; 3 Institute for Space Medicine and Physiology (MEDES), 1 Avenue du Professeur Jean Poulhes, Toulouse, 31400 France; 4 Maidstone Hospital, Maidstone and Tunbridge Wells NHS Trust, Hermitage Lane, Maidstone, ME16 9QQ UK; 5 Klinik für Anästhesiologie und Operative Intensivmedizin, Universitätsklinikum Köln, Kerpener Straße 62, Köln, 50937 Germany

## Abstract

Future space exploration missions will take humans far beyond low Earth orbit and require complete crew autonomy. The ability to provide anaesthesia will be important given the expected risk of severe medical events requiring surgery. Knowledge and experience of such procedures during space missions is currently extremely limited. Austere and isolated environments (such as polar bases or submarines) have been used extensively as test beds for spaceflight to probe hazards, train crews, develop clinical protocols and countermeasures for prospective space missions. We have conducted a literature review on anaesthesia in austere environments relevant to distant space missions. In each setting, we assessed how the problems related to the provision of anaesthesia (e.g., medical kit and skills) are dealt with or prepared for. We analysed how these factors could be applied to the unique environment of a space exploration mission. The delivery of anaesthesia will be complicated by many factors including space-induced physiological changes and limitations in skills and equipment. The basic principles of a safe anaesthesia in an austere environment (appropriate training, presence of minimal safety and monitoring equipment, etc.) can be extended to the context of a space exploration mission. Skills redundancy is an important safety factor, and basic competency in anaesthesia should be part of the skillset of several crewmembers. The literature suggests that safe and effective anaesthesia could be achieved by a physician during future space exploration missions. In a life-or-limb situation, non-physicians may be able to conduct anaesthetic procedures, including simplified general anaesthesia.

## Introduction

Significant plans have been drawn by government space agencies and private companies for manned spaceflights beyond low Earth orbit (LEO) in the coming years, with a focus on missions to Mars. Such flights have been termed space exploration missions (SEM). The latest National Aeronautics and Space Administration (NASA) mission design called for a 900-day mission for a crew of 6, with around 6 months spent in transit, each way, and 500 days on the Mars surface.^1^

These interplanetary missions will present great challenges to the field of space medicine.^2,3^ During the exploration of frontiers on Earth, human physiologic maladaptation, illness, and injury have accounted for more failures than technical or environmental factors.^4–6^ Beyond the immediate vicinity of Earth, there will be no possibility for the crew to return swiftly to the ground or to be assisted in real-time from Earth.^3,7^ Such space exploration will entail extreme isolation and therefore total crew autonomy.^2,4^

Among the expected severe medical conditions, surgical problems are of central concern, and will require anaesthesia,^4,8–13^ which currently represents a gap in space medicine knowledge.^12,14,15^ No human has ever required an anaesthetic procedure in space or shortly after returning to Earth. It is not appropriate to test protocols on healthy astronauts in space, and efficient ground models do not exist.^12,14,16^ The current contingency plan for any severe illness occurring in LEO includes rapid stabilisation in orbit and station evacuation.^2,17,18^ Therefore, it is likely that the first extra-terrestrial anaesthesia will be conducted during a SEM.

Researchers have extensively used space analogue environments (such as polar bases or submarines) to probe hazards, develop crew proficiency, validate medical technologies and countermeasures for prospective space missions.^2,3,5,19–21^ Studying medical care in these space analogue environments can provide predictive insight into the many factors that will impact healthcare delivery during future SEM.^3,5,22^

Our objective is therefore to conduct a literature review about anaesthesia in space analogue environments, to further our understanding of the challenges at stake and propose some possible solutions. We will present how various problems have been addressed in relevant settings, and discuss how this information could be applied to the unique environment of a SEM. The question of surgical preparedness, and the extent of the surgical procedures that the crew will be able to carry out are outside the scope of this work. This literature review will aim to provide important information for the design of the on-board healthcare system and protocols for future SEM, as well as clues for future research pathways to help close remaining gaps.

## Results

### Publications inclusion flow diagram

**Fig. 1:**
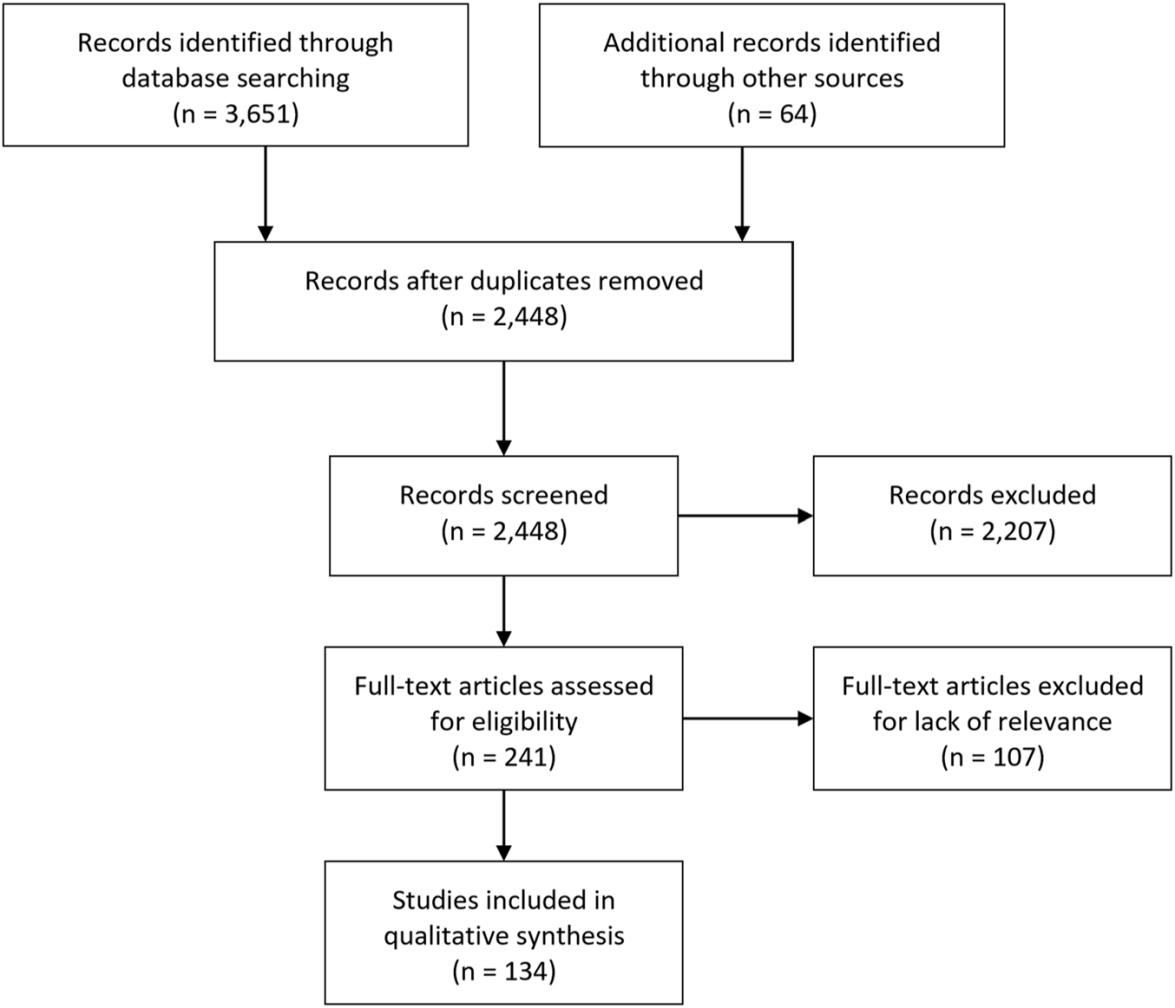
PRISMA flow diagram of publications reporting on anaesthesia in austere environments relevant to a future space exploration mission, published from January 2000 to December 2016

The Fig. 1 shows the results of the review process. We screened 2448 search results by title and abstract for possible inclusion. The full texts of 241 publications were assessed for eligibility. In total, 134 publications were included in the review, represented by 55 research articles, 31 reviews, 17 book chapters, 9 books, 9 reports, 5 clinical guidelines, 5 editorials and 3 case reports.

### Characteristics of the selected austere environments

A summary of the characteristics and limitations of the four selected environments is shown in Table 1.

**Table 1:** Summary of the characteristics and limitations of the selected analogue environments

	LEO	LMIC/humanitarian	Combat	ICE
Description	Use of LEO as analogue for interplanetary travel research	Resource-poor setting with lack of equipment and medical expertise	Out of hospital austere setting, high prevalence of severe trauma and blast injuries	Expeditions in environments such as: mountain, high altitude, polar, jungle, desert
Volume of surgical procedures^a^	Nil	High	High	Low
Technological and equipment constraints	++	+++	++	++
Human constraints	++	+++	++	++
Patient physiological alterations	++ secondary to exposure to microgravity	±	Soldiers are healthy individuals before injury	± (possible hypoxia, hypo/hyperthermia)
Limitations for applicability to SEM	No experience of surgery or anaesthesia. Lacks total isolation (option to evacuate, real-time telemedicine)	Does not provide microgravity and/or radiation exposures. Anaesthesia providers very different from astronaut population	Does not provide microgravity and/or radiation exposures. Pathology nearly exclusively traumatic. Well trained providers	Does not provide microgravity and/or radiation exposures

*LEO* low earth orbit, *LMIC* low and middle income country, *ICE* isolated and confined environment

+: present; ++: important; +++: major

^a^skill erosion is not expected in environments with a high caseload

LEO can be used as an 'in-space' analogue for interplanetary travel research.^23^ Orbital flights represent the closest analogue to SEM in terms of environmental exposure, but lacks experience of invasive medical procedures and isolation. Indeed, crews can communicate in real-time with the ground, benefit from telemedical support and can be readily evacuated in case of serious illness.^2,7,23,24^

Low and middle income countries (LMICs) are defined as those with a gross national income per capita, calculated using the World Bank Atlas method, of $12,235 or less.^25^ These countries are invariably afflicted by severe limitations in healthcare resources and skilled personnel.^26–31^ Several significant international endeavours such as the WHO Emergency and Essential Surgical Care programme, the Lancet Commission on Global surgery and the World Bank publication Essential Surgery in Disease Control Priorities in Developing Countries—3rd Edition (DCP3) have released guidelines and tools to improve safety and cost-effectiveness of surgery and anaesthesia in resource-poor settings.^28,32,33^ They have been defining 'the lowest common anaesthesia denominator', which is an appealing concept for future SEM where facilities and skills may be reduced to the bare minimum.^29^

Combat anaesthesia refers to the provision of anaesthesia by trained professionals or non-medics to soldiers and civilians in armed conflict environments. This setting is characterised by little to no infrastructure, limited logistical support, in remote or dangerous areas.^34–39^

Isolated and confined environments (ICEs) include a broad variety of places that present hostile and harsh physical conditions posing threats to human health and life.^5,23,40^ ICEs encompass a wide range of environments and medical specialties such as expedition, wilderness, mountain, diving, underwater, polar, sailing, aviation, jungle, desert, among others.^20,23,40–43^ In ICEs, micro-societies of scientists and explorers expose themselves willingly to such environments. In general, the teams include trained physicians or paramedics carrying a limited medical kit. Their ability to perform advanced medical care is sometimes so limited that ICEs have been described as 'fourth world' medicine.^42^

### Expected medical and surgical conditions

In austere environments, the precise anticipation of likely medical conditions is important because it influences the design of the health system (equipment, personnel and skills).^9,40,44^ In these environments, most surgical conditions are traumatic or infectious.^2,21,26,28,32,34,35,38–41,45–48^ Several sources have listed the surgical procedures which should be available anywhere at any time, as they are deemed essential.^32,39,49–51^

No human surgery has ever happened in space, and indeed astronauts have experienced very few events that could have required surgery. Station evacuations have occurred on three instances in the 1970’s and 1980’s for suspected appendicitis and dysrhythmias but were also hastened by psychological issues and crew conflicts.^4,22,52,53^

The likelihood of events requiring anaesthesia during SEM can be estimated to some extent from large case series from ground-analogue populations, military and civilian populations, and data gathered throughout the 140 person-years of cumulated spaceflight experience.^8–10,13^ For example, the risk of acute appendicitis has been estimated as 1–2 per 100,000 person-days, which would be equivalent to 1–2 cases every 45 years for a 6-person space crew.^8^ The cumulated risk of severe event requiring surgery may add up to one or more during a 900-day Mars mission.^4,8,9,19^ However, owing to the duration, remoteness, activities (more extra-vehicular activities—EVAs) of SEM, the exposure to the hazards of space will be different than in LEO and lead to various types of medical conditions.^13^ Overall, experts have estimated that the most significant risks for SEM are trauma, haemorrhagic shock^9,11^ and infections.^54,55^ Indeed, a loss of bone mineral density of 1–2% per month has been measured and increases the risk of renal stones and possibly of osteoporotic fractures after a few months into the flight.^2,3,56^ Although the risk of severe trauma is low in weightlessness, objects conserve their mass and therefore still carry kinetic energy when in movement.^11,15^ The vast number of EVAs planned during surface exploration will expose the astronauts to a high cumulated risk of traumatic accidents and hypobaric decompression sickness.^9,13,57^ The risk of infection is increased in space, due to space-induced immunosuppression and possible increase in bacterial virulence.^54,55^ The full list of expected conditions is summarised in Table 2, along with non-surgical illnesses, provided for reference.^9,13,57^

**Table 2:** List of expected surgical conditions, recommended procedures and medical illnesses during SEM, not including pregnancy-related conditions

1- Surgical conditions and procedures	2-Non-surgical conditions
1.1- Trauma	2.1- General medical conditions
Suturing laceration Tube thoracostomy Fracture reduction Irrigation and debridement of open fractures Fractures: external and internal fixation; use of traction Trauma laparotomy or Eescharotomy/fasciotomy Trauma-related amputations Skin grafting Burr hole Surgical airway	Minor trauma, sprains and strains Infections: pneumonia, cellulitis, gastroenteritis, urinary tract infection, corneal infection, latent viral reactivation Cardiovascular diseases: myocardial infarction, cardiac dysrhythmias Renal stones Psychiatric: depression, anxiety, sleep disorders Cancer
1.2- General surgical	2.2- Space-specific conditions
Drainage of superficial abscess Dental extraction, drainage of dental abscess Repair of perforations: for example, perforated peptic ulcer Appendectomy Bowel obstruction, colostomy Gall bladder disease, including emergency surgery Relief of urinary obstruction: catheterisation, suprapubic cystostomy Treatment of renal stone including nephrostomy Hernia, including incarceration Drainage of septic arthritis Biopsy	Cardiovascular deconditioning, orthostatic intolerance Radiation exposure Visual impairment and intracranial pressure syndrome Space motion sickness Environmental exposure including hypobaric decompression sickness, toxic atmosphere, hypothermia/heat stroke, planetary dust

Adapted from.^13,32,50,51,57^

The provision of anaesthesia under these circumstances will be restricted to procedures which are absolutely essential for the saving of life or limb.^29,31,58^ Non-operative treatment may also be chosen, for example for uncomplicated appendicitis.^4,10^ Of note, prophylactic removal of the appendix and/or gall bladder is being considered.^4^

### Causes of death

The analysis of the root causes of death in austere environments provides some insight into the most critical aspects of healthcare safety, and helps identify potential strategies for improving outcomes.^35,59^

Perioperative mortality is usually due to a combination of factors related to patients, surgery, anaesthesia and general management.^31,48,59^ In LMICs, shortages and misdistribution in the anaesthetic workforce, as well as deficits in health infrastructure have been consistently correlated to mortality.^28,30,31,60–63^ Most often, perioperative deaths in LMICs are related to acute anaemia or septic shock, frequently hastened by pre-existing morbid conditions.^31,48,59,61,64^ In the combat environment, haemorrhage remains the most common cause of death.^35,36,45^ In these circumstances, the prevention and prompt correction of major blood loss is critical and requires the availability of blood products.^31,36,49,64,65^ Postoperative infections are usually prevented with antibiotic prophylaxis and sterilization of instruments,^31,66^ although the risk of post-operative infection persists in situations such as trauma with contamination, hollow viscus perforation or intra-abdominal abscess and will require full course of antibiotics.

Less often, deaths occur as a direct consequence of anaesthesia. In LMICs and elsewhere, preventable anaesthesia mortality stems from two main factors: hypoxemia and hypotension, which are primarily related to failed intubation and induction of anaesthesia in the presence of hypovolaemia.^29,31,60,64^ Anaphylaxis, aspiration, cardiac events and medication interactions are also potentially life-threatening problems.^31,60^

The safety objective for a mission to Mars has been defined by some experts at 3% of individual risk of death per year.^9^ A significant portion of this risk is related to spacecraft failure, the rest being represented by death from trauma and medical illness, which amounts to approximately 0.24% per individual and per mission.^9^ Trauma, infections, haemorrhage, radiation sickness and cardiovascular events represent the most likely causes of death.^9,11,55,67–70^ The risk of infection appears increased in space, due to several factors related to immunosuppression, possible increase in bacterial virulence, and presence of particles in suspension.^55^ The haemodynamic tolerance to blood loss or sepsis is expected to be poor due to changes in volaemia and cardiovascular performance experienced after exposure to microgravity.^67,71^

In LMICs, surgeries with a low probability of success and a high probability of death are often not attempted.^31,32,48,60,72,73^ Similarly, during a SEM, the crew must prepare for non-survivable illnesses or injuries that will exceed the local treatment capability.

### Medical skills of anaesthesia provider(s)

To achieve the minimum standard in patient safety, models for surgical and anaesthesia training have been developed.^29,31,32,39,48,49,60,74^ These models are organised in several layers of complexity, with successive strata allowing increasingly complex procedures. In these models, the core components provide basic resuscitative and primary trauma capacity that do not require extensive equipment or skills.^30,31,39,60,75^ The second level allows treatment of most life-threatening conditions and includes spinal and ketamine-based anaesthesia, laparotomy, amputation, closed and open orthopaedic surgery.^31,49^ This requires physician-level skills but not necessarily that of an anaesthetist, to provide full resuscitation and general and spinal anaesthesia.^60^ The third level requires specialist-level skills to deliver prolonged multi-organ support.^31,60^ In these models, medical services in scarce environments focus on primary care rather than on more advanced medical and surgical care, which likely saves more lives.^28,31^

In LMICs, the shortage of physicians is such that it is common for non-doctors (nurses, anaesthetic officers, clinical assistants…) to carry out anaesthesia and surgery, many of whom have little medical background and are trained 'on the job'.^26,28,29,47,76^ Their ability to deal with complex cases remains limited.^27–29^ A key difference with future SEMs is represented by the high number of patients treated by anaesthetic providers in LMICs.^27,29,30,77^ Consequently, skill retention is less an issue in LMICs, while representing a huge challenge in future long-term SEMs.

Simulation plays an important role in the acquisition of anaesthetic and non-technical skills, both for doctors and non-doctors, as demonstrated in many studies looking at patient outcomes.^78,79^ Models for simulation in low-resources settings and distance learning of anaesthetic skills have been proposed.^76,78–81^ 'Just-in-time' training allows practitioners to gain or refresh skills on-the-spot, for example in case of unexpected scenarios.^82^ We retrieved the case of a spinal anaesthesia delivered in Antarctica by a non-anaesthetist with remote support.^83^

In combat environments, personnel with various levels of medical skills deal with trauma casualties, often severe and in large numbers.^38,39,45,49,72^ Nurse anaesthetists have been and remain the main providers of anaesthesia care to military personnel.^36,49^ The observation that over 90% of deaths happen before the wounded reach a medical facility has led to efforts to broaden medical training to non-medical personnel on the field, including for advanced procedures such as thoracocentesis and surgical airway.^39,45,84^

The current International Space Station (ISS) programme requires the presence on-board of a crew medical officer, who is not necessarily a physician.^2,18,85^ The ideal profile for the crew physician on future SEM is still debated, due to the uniqueness of the operation.^13,86^ The best physician profile for a SEM could be an emergency medicine doctor with additional training in surgery and wilderness medicine.^13,86^ Importantly, the crew doctor will spend most of his time on non-medical tasks, which increases further the complexity of his training during mission preparation.^12^

The crew physician will need to have a broad knowledge base, to be competent in basic surgical skills and in the management of the critically ill and injured.^13,85,86^ One of the most important qualities will be flexibility and thus the ability to improvise in medical scenarios that may have been unseen before.^13,40^

Most likely, a single physician will oversee both the surgery and the anaesthesia.^28^ Skills redundancy will be critical to enhance crew safety, especially if the physician himself becomes ill, injured, incapacitated or dies.^18,85,87^ In this situation, it has been suggested that non-physicians could perform advanced medical care.^12,87^ It appears advisable to train several crewmembers to manage the most common emergencies, for example matching the first level of competency of the WHO or DCP3 models.^31,32,77^

Significant advances have occurred in recent years in the field of artificial intelligence in medicine, that offers the promises of more effective monitoring, improved disease detection and development of efficient decisions support systems.^2,88^ Autonomous diagnostic systems, closed-loop automated anaesthesia or other decision support systems could simplify training requirements and improve patient safety.^88–90^

### Non-clinical skills, behavioural health and performance

Prolonged exposure to factors such as stress, workload, fatigue, social isolation, altered lighting conditions and circadian cues all contribute to degraded performance, both on ICEs and in space.^82,91,92^ The negative psychological response to living in ICEs include mild cognitive impairment, time-sense disturbances, motivational decline, sleep disorders, psychosomatic symptoms, anxiety, depression and social conflicts.^5,6,22,52,91,92^ Maintaining crew behavioural health and performance has arose as one of the most challenging aspects of prolonged stays in ICEs.^5,6,22,82,91,92^

Practical concepts aimed at improving operational performance both in austere environments and in space have been proposed. Schematically, they revolve around three aspects of performance, all non-specific but directly applicable to medical skills: correct crew selection, training prior to the mission, and skills acquisition and maintenance during the mission.^82,91^ Identifying 'the right stuff' for an unprecedented challenge such as a SEM, both at the individual and the team level, and then maintaining mental health and crew cohesion during the entire flight will be a key component of mission success.^5,22,52,91^ Medical and psychological standards for crewmember selection are likely to be extremely restrictive owing to limitations in medical care and support.^22,52,91^ In austere environments, many non-clinical skills of the physician contribute to healthcare safety.^31,36,49,73^ These can be divided into personnel skills (e.g., team coordination, communication, or logistics) and technical skills (e.g., troubleshooting equipment, use of safety equipment, or orientation).^5,40,82,91^

### Medical kits

In austere environments, a desirable situation for medical support is to match the equipment and personnel competencies to deal with the most likely medical conditions.^40,44^

The basic equipment required for safe anaesthesia need not be elaborate: a basic mechanical ventilator, monitoring including a pulse oximeter and capnography, airway equipment and a restricted range of drugs.^30,31,51,58,60,93^ In the most deprived settings, limited kit, even though not ideal, can be adapted to maximise patient care.^29^ It has been suggested that the monitoring setup could even be reduced further to continuous clinical monitoring and a pulse oximeter, for a solution that is truly achievable in the poorest settings.^29^ This is less relevant to future SEM since acquisition cost may be less important than the mass of the equipment. Oxygen is desirable but unavailable in most LMICs.^29,30,58^ In the absence of a mechanical ventilator, manual ventilation can be handled by a non-physician, who has usually been trained on-the-job.^60,75^ The equipment for local and regional anaesthesia (RA) is much more limited, which makes it very desirable in resource-poor environments, but only when anaesthesia providers are competent.^15,34,61–63^

The care of a surgical patient requires a range of support services and equipment that extend well beyond anaesthesia, such as running water, electricity, surgical equipment and sterilization means, personal protective equipment, laboratory work, imaging equipment, ideally continuous oxygen and blood products.^26,28,30,31,45,49,51^ Sterilization of surgical instruments in austere environments is challenging, but simple dry heat or antiseptic methods are acceptable.^31,66^

Checklists are a simple and cost-effective way to improve patient safety.^29,94,95^ They are particularly useful where expertise is limited, because they help with memory recall and clarify the minimum expected critical safety steps in a complex process.^31^

The current ISS medical kit does not allow for general anaesthesia (GA) or prolonged organ support and will need to be profoundly updated for a SEM.^8,18,87^ The design of the medical kit must balance crew skills with constraints in volume, weight, power requirements against the load of expected medical conditions, which partly depends on the mission profile (e.g., mission duration, number of EVAs…) and crew size.^9,44,87^

During SEM, restrictions in storage and up-mass and the impossibility to re-supply may lead to shortages in tools and consumables. On-demand 3D printing of equipment is promising.^96^ Methods to ensure drug stability during the mission must be developed and validated.^97^ The expected lack of blood products could be mitigated using fresh whole blood transfusion, similar to the concept of 'walking blood bank' in combat medicine.^98^ This would imply that blood compatibility could become a selection criteria.^45,49,65^ Ultrasonography is likely to remain the leading imaging modality in future SEM.^13^ It can be used for a variety of tasks related to anaesthesia and surgery, such as nerve localisation, assessment of volaemia and cardiac function, line placement, and assisting external fixation of fractures.^13,99,100^ Acquisition of several critical skills appears shorter with ultrasound, even in novices. For example, with a standardised teaching program, ultrasound-guided central venous catheter insertion can be learnt by non-experts after less than ten procedures^101^

### Telemedicine

Telemedicine relies on remote communication technologies to allow experts to provide diagnosis and/or therapeutic advice for patients situated in an isolated place.^2,20,21,24,41,43,78,102^ Real-time communication is not mandatory for telemedicine. For example, augmenting remote consultation by transmitting X-rays, ultrasound images and digital photographs by email has proven very valuable.^40,41^ The proof of concept of a remotely administered general and spinal anaesthesia in real-time have been demonstrated.^83,103^ Telemedicine is already extensively used in spaceflight for remote diagnosis and treatment, monitoring and training of astronauts.^7,18,23,24^ Real time telecommunication will not be available during a mission to Mars, with delays ranging from 5 to 20 min each way, depending on the relative position of the planets.^4,21^ Concepts of delayed asynchronous tele-guidance for surgery on Mars have been proposed.^21^

### Preoperative assessment

The preoperative assessment of a patient in difficult environments should insist on the assessment of the risk of aspiration and difficult airway, and estimation of the recent fluid loss from bleeding, vomiting, diarrhoea, anorexia or other.^31,60^ Most patients are hypovolaemic due the ongoing pathological process requiring surgery (trauma or infection) or secondary to dehydration.^38,40^

In space, microgravity affects most physiological systems.^71,104^ The loss of the gravitational stimuli profoundly alters the cardiovascular system, which rapidly becomes unable to respond efficiently to challenges such as orthostatism or blood loss.^67,71,105^

**Fig. 2:**
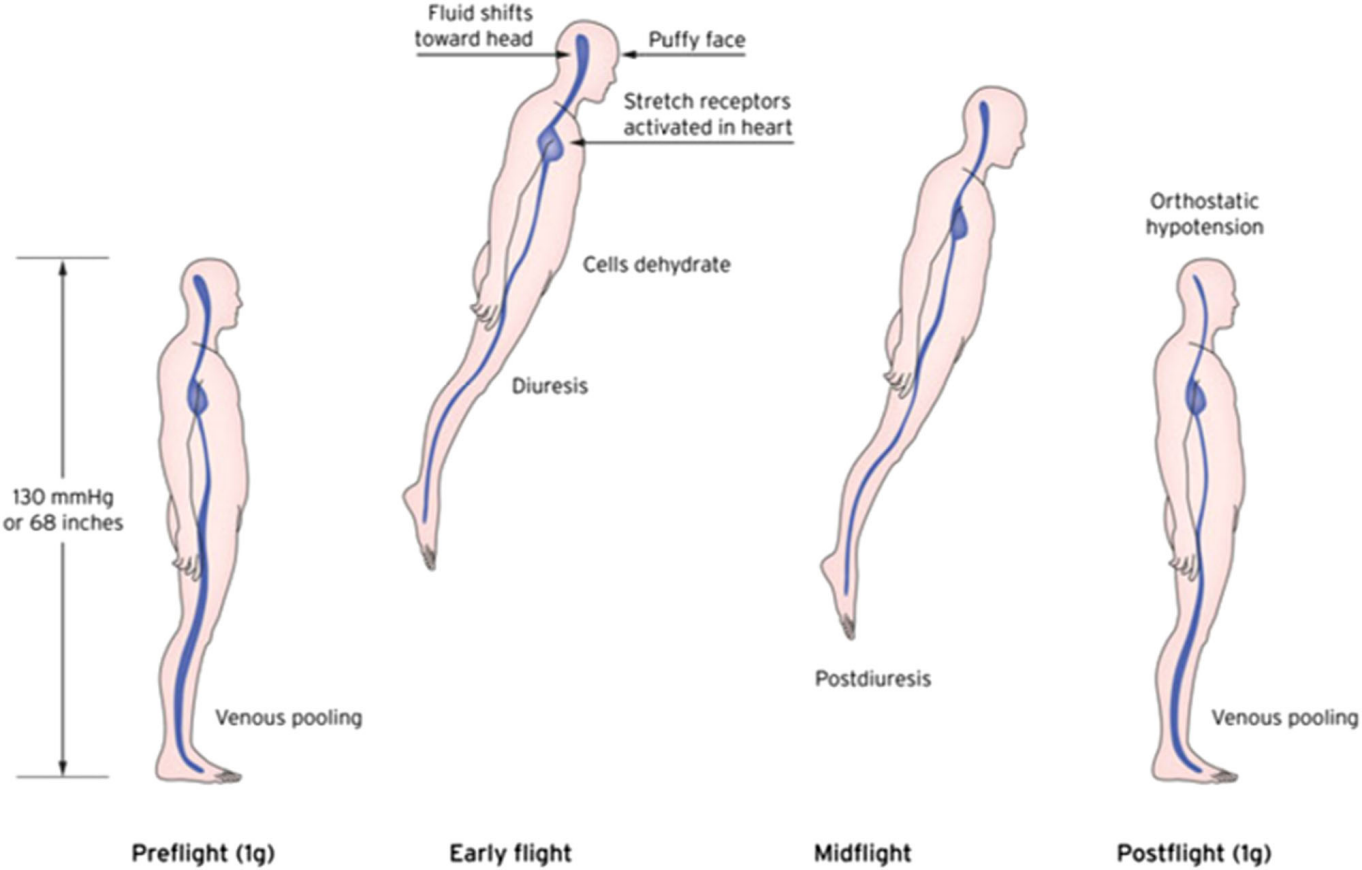
Fluid shift in space, and its involvement in post-flight orthostatic intolerance. Immediately after entering weightlessness, a portion of the blood volume shifts towards the upper body, leading to the 'puffy face' and 'chicken legs' syndrome. Post-flight, the combination of hypovolaemia and hypo-reactivity of the cardiovascular system commonly leads to orthostatic hypotension. Reproduced with permission from Gunga^71^

The cardiovascular profile of a microgravity-exposed individual is marked by a 15–20% hypovolemia, altered baroreflex and systemic vascular resistances, changes in adrenergic receptors, mixed systolic-diastolic cardiac dysfunction, all leading to up to 20% decrease in exercise tolerance.^12,67,71,104,106^ These factors expose the astronaut to a significant risk of cardiovascular collapse during induction of GA and mechanical ventilation.^12,14,107^ The cardiovascular system faces its biggest challenges of spaceflight upon return (Fig. 2). Orthostatic intolerance affects over 80% of ISS crewmembers and is regarded as one of the most serious cardiovascular problems upon return to Earth.^2,67,71,104^ The extent of the cardiovascular alterations in partial gravity (such as on Mars) and the level of gravity required to prevent these effects are currently unknown.^71^

The patient’s volaemic status and cardiac function should be assessed with physical examination and ultrasound, and if possible optimised before induction. The expected volume of ultrasound procedures required to train a non-expert at these techniques can be estimated from the literature. Several days of training and 25 supervised procedures were necessary to train nurses to perform ultrasound measurement of the aorta and inferior vena cava diameters in a resource-limited setting.^108^ At least 10 h of mixed didactic and scanning training and 45 procedures may be required for emergency physicians to become proficient in focused cardiac ultrasound.^109^ In the microgravity-exposed patient, it may be advisable to administer a low dose of vasopressors (alpha-agonists) preventively before induction, especially if GA or mechanical ventilation are anticipated.^12,14,87^

### General and regional anaesthesia

#### General anaesthesia

In LMICs, where medical expertise and airway equipment is lacking, a vast proportion of procedures (up to 90%) are carried out under GA without intubation.^26,47^ Oro-tracheal intubation requires extensive training.^110–112^ It has been estimated that over 50–60 conventional oro-tracheal procedures were necessary for a novice to reach a 90% success rate.^110,113^ With videolaryngoscopy, this number may drop to less than ten procedures.^112,114^ Even if the majority of the literature identified steeper learning curves and shorter intubation time with videolaryngoscopy, negative studies exist and this technique still requires appropriate training.^111,112^ Capnography should be used to confirm successful airway insertion.^115^ In one study, novices could achieve a 80% success rate in bag-and-mask ventilation after a median of 25 procedures.^116^

Ketamine is the drug of choice for non-anaesthetic trained doctors, because it is cost-effective and relatively safe.^31,38,60,77,117^ It is used extensively in adult and paediatric populations in military anaesthesia, LMICs and during disaster relief.^26,38,46,47,77,117–120^ Properly trained individuals can use propofol, although it is a poor choice for induction in patients with shock, even after fluid resuscitation.^38^

In 2002, a NASA working group stated that the safe delivery of anaesthesia in space could be achievable.^12^ Inhaled anaesthesia is not an option in the closed environment of a spacecraft, because of issues of cabin pollution and because vaporisers would be unreliable in reduced gravity.^12,15,107^ Among intravenous agents, ketamine appears to be the safest choice.^12,14,57^ Of note, ketamine-based anaesthesia has been performed on 22 monkeys shortly after spaceflight.^121^ Hallucinations and emergence phenomena can be mitigated with premedication and overall are a lesser concern in a life-or-death situation. Gastric motility is slower in space, at least during the first days, possibly for longer, so systematic rapid sequence induction is advisable. Succinylcholine is contraindicated after exposure to microgravity because of changes in the neuromuscular junction.^12,14,107^ Both operator and patient must be restrained if conventional laryngoscopy is to be attempted in weightlessness, otherwise supraglottic devices are an option.^122–124^ Videolaryngoscopes have been tested in a Mars analogue simulation.^87^

#### Regional and perimedullar anaesthesia

The use of RA in difficult environments is appealing, because it requires fewer preoperative, intraoperative and postoperative resources.^15,34,38,62,94^ Indeed, regional and perimedullar anaesthesia (spinal anaesthesia in particular) are often the preferred choice where expertise is available, but are otherwise virtually non-existent.^26,28,34,38,47,48,62,64^ They are regularly delivered by non-anaesthetists, demonstrating that they can successfully be trained to RA techniques in austere environments.^38,62,83,125–127^ Most limb surgery is feasible with only 3 blocks (axillary brachial plexus, femoral and sciatic blocks).^12,15,57,128^ The use of ultrasound for RA has accelerated the training of anaesthesia residents and improved success rates.^12,57,128^ In ultrasound-guided nerve blocks, anaesthesia residents commonly require a minimum of 10–15 procedures per block to achieve a 90% success rate.^129^ Many additional techniques such as intravenous RA or haematoma blocks can be of value in difficult environments.^12,38,120^ In LMICs, intra-abdominal surgery such as caesarean section are commonly performed under RA alone, after infiltration of the abdominal wall by large volumes of lidocaine.^60^

For future SEM, RA is an extremely interesting and safe option despite its limitations, and efforts should be made to integrate RA into the crew physician’s skillset.^12,15,57^ The absence of sedation and shorter recovery times will enable a faster return to full operations and minimise the impact on the mission, in an environment where everyone will have a unique and valuable skillset. The safety and efficacy of perimedullar anaesthesia in weightlessness or in partial gravity is unknown but concerns have been expressed about the effect of the sympathetic block on a microgravity-exposed patient.^12^

### Choice of anaesthetic technique

The general approach for choosing an anaesthetic technique in a difficult environment depends on several factors: patient’s condition, training and experience of the anaesthetist and surgeon, availability of drugs and equipment, degree of urgency, presence of a full stomach, and finally patient’s preference.^60^ Anaesthesia providers with limited experience should limit themselves to a small number of safe, widely applicable techniques, to improve familiarity and confidence through regular practice.^75^ Dobson suggested that because of limited skills and supplies, GA use should be minimised whenever possible.^75,94^

During SEMs, in the absence of strong evidence, it appears sensible to formulate choices based on a worst-case scenario approach and consider that astronauts requiring surgery will be severely deconditioned, hypovolemic, at risk for arrhythmias, difficult to intubate, intolerant to succinylcholine, have a full stomach, and be managed by non-medical personnel with limited training, if the crew medical doctor is incapacitated or dead.^12,57^ Overall, we argue that RA should be attempted whenever possible.^12,15^ When not suitable or in case of failure, GA will be necessary. We recommend to implement a limited number of simplified intravenous anaesthesia protocols that could be narrowed down to two options only: conscious sedation (for procedural anaesthesia, peripheral surgery and superficial trunk surgery) and GA with endotracheal intubation (for head, face and deep trunk surgery).^12,57,87^

### Postoperative care

The most important aspects of postoperative monitoring do not rely on complex equipment: airway patency, haemodynamic and respiratory stability, urine output, warmth of peripheries and pain control.^31,60^ Following surgery, the most severe patients will require sustained invasive support, which occurs typically in an intensive care unit. In LMICs, such facilities are excessively limited.^47^

A key focus of the postoperative period involves pain control, which is more difficult and inconsistent in austere environments.^120^ The Wilderness Medical Society guidelines propose a pyramidal approach to pain management in austere environments, with simple physical and comfort measures representing the basis of the management, before any escalation of care. The ideal medication for austere environments (compact, non-sedating, long shelf-life, with multiple routes of administration, minimal side effects and a wide spectrum of use) does not exist.^36,120^ Potent drugs with harmful side effects (narcotics, ketamine) are reserved for the most severe pain, only after safer and less-invasive therapies have been considered. Local and regional anaesthesia are valid choices for pain relief, provided the caregiver is accustomed to these techniques.^34,120,130^

Postoperative care and pain control during SEM should follow general Earth-based guidance, with the necessary adjustments aiming at improving crew recovery and limiting resource utilisation.^57,120,131^ The provision of critical care during SEM is beyond the strict scope of this review, but if we extrapolate from the current ISS capabilities, it is unlikely that the crew will have the capacity to provide prolonged organ support of one or several critically ill patients following surgery or resuscitation.^12,18,87^

## Discussion

Imray (2015) anticipated that future developments in healthcare in difficult environments will be determined by the needs of modern day explorers.^40^ He argues that travellers will encounter 'environments where physiological and geographical extremes necessitate prompt and innovative approaches to rescue, medical care, and transportation'.^40^ Future SEMs perfectly illustrate this statement. However, medical preparedness for SEM is difficult to achieve as experimentation in space is constrained by access and operational resources, and because of the small sample size and low incidence of medical conditions. The need to find relevant terrestrial substitutes is driven by extraordinary demands for mission success.^5^ Among medical procedures, the delivery of anaesthesia currently represents a gap in knowledge.^12^ Therefore, we have analysed an extensive set of topics surrounding the practice of anaesthesia in environments relevant to SEM, with the objective of closing this gap.

The summary of our results is shown in Table 3. The literature seems to indicate that non-anaesthetists, and, as a last resort, non-physicians, could potentially provide effective and relatively safe anaesthetic procedures during future SEM, provided that they receive the appropriate training during the preparatory phase. It makes little doubt that astronauts, with their extensive skill-set, cognitive aptitudes and ability to deal with extreme stress, are among the best candidates to overcome such a challenge. Besides the very unique context of a spaceflight, the findings of this research could also benefit Earth-based initiatives and the general public, by improving anaesthesia delivery and safety in remote and resource-poor settings.

**Table 3:** Summary of findings and recommendations

1. The equipment, protocols and training programme for anaesthesia need to be developed and designed alongside all related medical sub-specialties, in particular surgery.
2. The basic principles of safe surgery (safety checklist, prevention of surgical site infection, confirmation of site and procedure, etc.) must be guaranteed. Checklists should be provided for all the essential steps, including general surgical safety (following the WHO model), preoperative assessment and anaesthetic procedures.
3. The personnel in charge of delivering anaesthesia require specific training.
4. Skills redundancy will be an important safety parameter. Several crewmembers must be trained to achieve at least a basic level of competency (similar to WHO level 1).
5. Most expected conditions requiring anaesthesia are traumatic and infectious. The availability of blood products or substitutes is expected to improve the survivability of severe bleeding.
6. Non-anaesthetists can perform anaesthesia. Physicians are preferred, but as a last resort (life-or-limb situation), non-physicians could attempt to perform advanced medical care including surgery and anaesthesia.
7. A restricted set of equipment can be sufficient. The strict minimum set of required equipment for anaesthesia is small, but caring for a surgical patient requires extensive equipment and consumables that spans well beyond.
8. The number of available anaesthesia protocols should be minimised, and efforts should be made to simplify them.
9. Ketamine appears to be the most suitable intravenous anaesthetic agent for general anaesthesia and procedural sedation. Videolaryngoscopes could be the preferred equipment for endotracheal intubation.
10. Regional anaesthesia is an appealing option for limb surgery. A limited number of blocks are sufficient. Ultrasound guidance accelerates training and improves success rate.

While the analysis of space analogue environments is important given the restricted access to space, no substitute can fully replicate the uniqueness of a future SEM, where a self-reliant restricted crew will be exposed to exceptional challenges and risks, some of which are impossible to foresee.^5^ Space analogues are only simulations of greater or lesser fidelity along varying dimensions of interest.^5,23^ We have not included research carried out in highly controlled simulation centres, because very few studies have explored the question of anaesthetic care provided by non-medical personnel, but also because the very attributes of the environment that have the greatest impact on performance are removed in simulation studies (e.g., real danger, uncontrolled events, situational ambiguity, or the interaction with the extreme environment itself). Bishop has argued that the value of this research was very limited once these features were compromised.^5^

While this review provides useful clues regarding some critical aspects of anaesthesia in space, several factors remained unexplored and warrant further research. More research needs to be done to define the ultimate skillset of the astronaut physician, design tools to prevent skills erosion during the flight and address the question of skills redundancy.^5,13,22^ Designing the on-board medical kit will take place in parallel, and will partly be driven by anaesthetic and surgical capability and engineering requirements.^8,18,44^ A return to gravity is increasingly difficult with increasing flight duration.^71^ More research is needed to investigate the synergistic effects of prolonged exposure to space-derived stressors and partial gravity on human systems, and to resolve some contradictory findings.^2,71,132^

## Conclusion

Future spaceflight medical systems must permit a well-trained medical officer to autonomously provide care for the crew during the mission. Many considerations beyond the specific illness or injury will influence the outcome, including environmental factors, communications, supplies, crew preparation, skills redundancy and teamwork. Preparation for the management of surgical conditions is only in its infancy, but safe and efficient anaesthesia could theoretically be achievable.

## Methods

First, we established the list of the relevant questions that the literature review had to address. These questions explore various aspects of anaesthesia in austere environments and represent potential gaps in knowledge and/or technology for delivering anaesthesia during a prospective SEM. The list covers aspects such as medical training, the content of the medical kit, expected scenarios, physiological changes relevant to anaesthesia, etc. It was established from exploring reference anaesthesia textbooks,^133,134^ as well as from the authors’ expertise. The Table 4 summarizes the final list of 12 questions.

**Table 4:** List of questions used in the literature review

Category	Questions
Usefulness of analogues	1. What characteristics of each selected austere environment are relevant to a SEM?
Expected conditions	2. What medical and surgical conditions are encountered in austere environments and expected during a SEM?
	3. What factors contribute to patient death in austere environments and a SEM?
Medical skills	4. What is the profile and medical skills of anaesthesia providers in austere environments? What medical skills are recommended for a SEM?
	5. What non-clinical skills are important for healthcare delivery in austere environments? How can human behaviour and performance be optimised?
Medical kits	6. What equipment is necessary or optional for anaesthesia in austere environments and during a SEM?
	7. How is telemedicine used for healthcare in remote environments? How could it be used during future SEM?
Pre, per and postoperative management	8. How is the patient assessed and resuscitated before receiving an anaesthetic procedure? What are the specificities of the physiology of the microgravity-exposed patient?
	9. How is general anaesthesia administered in austere environments? How is the airway managed?
	10. What is the role of regional and perimedullar anaesthesia in austere environments? What blocks are recommended?
	11. What considerations are important for choosing the most appropriate anaesthetic technique in austere environments?
	12. How is the patient managed in the post-operative period in austere environments? What are the guidelines for pain control?

They explore various aspects of anaesthesia in austere environments and during a future SEM, and correspond to potential current gaps in space medicine knowledge and/or technology. Refer to text for explanations on how the list was established.

Secondly, we selected space analogue environments, characterised by the presence of up to four types of constraints that match those of a SEM:^5,12,23,30,40^

1. Environmental challenges, represented by extreme, hostile or uncontrolled conditions and physical and social isolation and confinement. Some of these settings also heavily rely on technological substitutes for life support (e.g., polar or underwater stations).
2. Inadequate resources: limited equipment and consumables.
3. Inadequate medical skills: care provided by non-medical specialists or non-physicians.
4. Difficulties in evacuation, because of distance, logistics, or hazards.

The four categories of environments that we selected are: spaceflight in LEO, LMICs, the combat environment and finally ICEs, such as polar bases in Antarctica, submarines, or remote expeditions.

Then, on 1 March 2017, we conducted a literature search on anaesthesia in the selected environments for the period 2000 to 2016, in the PubMed, Scopus, Google Scholar and Cochrane databases. Research articles, reviews, case reports, guidelines and consensus statements, books and book chapters in the English language were evaluated. The full search queries are provided in supplementary information. We have also examined publications from space agencies (the NASA Technical Report Server), military (the US Defense Health Board, the Committee on Tactical Combat Casualty Care, the UK Royal Army Medical Corps association) and humanitarian, non-governmental organisations and professional bodies: the International Committee of the Red Cross, the WHO, the World Federation of Societies of Anaesthesiologists, the DCP3, the 2011 American Society of Anesthesiology Guidelines, the 2014 Lancet Commission on Global Surgery. Duplicate findings were removed.

We screened 2448 search results by title and abstract for possible inclusion. The full texts of 241 publications were assessed for eligibility.

Finally, all authors screened the search results by title and abstract and compiled an inclusive list of potential articles of interest. All articles that were deemed suitable by at least one author were included for full review. All authors independently read all final selected articles and extracted any information relevant to the pre-defined questions.

## Electronic supplementary material

Supplementary Methods

References sorted by topic
