# Supplementary material for: Anaesthesia in austere environments: literature review and considerations for future space exploration missions
Source: NPJ Microgravity. 2018 Feb 23;4:5. doi: 10.1038/s41526-018-0039-y (PMC5824960; doi:10.1038/s41526-018-0039-y)
Supplement: Supplementary file 1 — Supplementary Methods [file 41526_2018_39_MOESM1_ESM.docx]

# Supplementary Methods

Article: Anaesthesia in Austere Environments: Literature Review and Considerations for Future Space Exploration Missions, by Komorowski et al.

Search queries for the review:

1. Pubmed: ("anaesthesia"[All Fields] OR "anaesthesia"[MeSH Terms] OR "anaesthesia"[All Fields] OR "anaesthetic"[all fields] OR "anesthetics"[all fields]) AND ("austere"[All Fields] OR "isolated environment"[all fields] OR "disasters"[all fields] OR "expedition"[all fields] OR "wilderness"[all fields] OR "developing country"[all fields] OR "relief work"[all fields] OR "combat"[all fields] OR "space medicine"[all fields] OR "space exploration"[all fields] OR "war"[all fields] OR "altitude"[all fields] OR "mountain"[all fields]) AND "humans"[MeSH Terms] AND ("2000/01/01"[PDAT] : "2016/12/31"[PDAT]). This search query returned 708 results.
2. Scopus: (TITLE-abs-key(anesthetic) OR TITLE-abs-key(anaesthetics) OR TITLE-abs-key(anaesthesia) or TITLE-abs-key(anaesthesia)) and (TITLE-abs-key(war) OR TITLE-abs-key(combat) OR TITLE-abs-key(space medicine) OR TITLE-abs-key(space exploration) OR TITLE-abs-key(relief work) OR TITLE-abs-key(austere) OR TITLE-abs-key(isolated environment) OR TITLE-abs-key(expedition) OR TITLE-abs-key(altitude) OR TITLE-abs-key(mountain) OR TITLE-abs-key(developing country) OR TITLE-abs-key(disasters) OR TITLE-abs-key(wilderness)) AND PUBYEAR > 1999 AND ( LIMIT-TO(SUBJAREA,"MEDI" ) ) AND ( LIMIT-TO(LANGUAGE,"English" ) OR LIMIT-TO(LANGUAGE,"French" ) ). This query returned 1,943 results.
3. Google scholar: anaesthesia "isolated environment" OR war OR combat OR expedition OR austere OR disaster OR "relief work" OR humanitarian OR "developing country" OR mountain OR wilderness OR microgravity OR "space medicine" -pediatrics -obstetrics -pain. This search query returned 25,100 results, of which we screened the first 1,000, sorted by relevance.
