# Supplementary material for: Anaesthesia in austere environments: literature review and considerations for future space exploration missions
Source: NPJ Microgravity. 2018 Feb 23;4:5. doi: 10.1038/s41526-018-0039-y (PMC5824960; doi:10.1038/s41526-018-0039-y)
Supplement: Supplementary file 2 — References sorted by topic [file 41526_2018_39_MOESM2_ESM.pdf]

	Reference	LEO*	LMIC*/Humanit.	Combat	ICE*	SEM*	Other
1	Mars Architecture Steering Group. Mars Design Reference Architecture 5.0. (2009).					1	
2	Doarn, C. R., Williams, R. S., Schneider, V. S. & Polk, J. D. Principles of Crew Health Monitoring and Care. in *Space Physiology and Medicine* (eds. Nicogossian, A. E. et al.) 393–421 (Springer New York, 2016). doi:10.1007/978-1-4939-6652-3_15	1					
3	Nicogossian, A. E. The Environment of Space Exploration. in *Space Physiology and Medicine* (eds. Nicogossian, A. E. et al.) 59–94 (Springer New York, 2016). doi:10.1007/978-1-4939-6652-3_2	1				1	
4	Ball, C. G. *et al.* Prophylactic surgery prior to extended-duration space flight: Is the benefit worth the risk? *Can. J. Surg.* **55,** 125–131 (2012).					1	
5	Bishop, S. L. From Earth Analogues to Space: Learning How to Boldly Go. in *On Orbit and Beyond* (ed. Vakoch, D. A.) 25–50 (Springer Berlin Heidelberg, 2013). doi:10.1007/978-3-642-30583-2_2	1			1		
6	Institute of Medicine (U.S.), Committee on Creating a Vision for Space Medicine during Travel Beyond Earth Orbit, National Academy of Science & Board on Health Sciences Policy. *Safe passage astronaut care for exploration missions*. (John R. Ball, Charles H. Evans Jr., 2001).	1				1	
7	Descartin, K., Menger, R. & Watkins, S. D. *Application of Advances in Telemedicine for Long-Duration Space Flight*. (NASA JSC, 2015).	1				1	
8	Campbell, M. & Billica, R. Surgical capabilities. in *Principles of Clinical Medicine for Space Flight* 123–137 (Springer, 2008).	1					
9	Comet, B. *et al.* *MARSTECHCARE, necessary biomedical technologies for crew health control during long-duration interplanetary manned missions*. 151 (ESTEC, 2002).	1				1	
10	Drudi, L., Ball, C. G., Kirkpatrick, A. W., Saary, J. & Grenon, S. M. Surgery in Space: Where are we at now? *Acta Astronaut.* **79,** 61–66 (2012).	1				1	
11	Kirkpatrick, A. W. *et al.* Severe traumatic injury during long duration spaceflight: Light years beyond ATLS. *J. Trauma Manag. Outcomes* **3,** 4 (2009).	1				1	
12	Komorowski, M., Fleming, S. & Kirkpatrick, A. W. Fundamentals of anesthesiology for spaceflight. *J. Cardiothorac. Vasc. Anesth.* (2016). doi:10.1053/j.jvca.2016.01.007	1				1	
13	Kuypers, M. I. Emergency and Wilderness Medicine Training for Physician Astronauts on Exploration Class Missions. *Wild Env. Med* **24,** 445–449 (2013).					1	
14	Agnew, J., Fibuch, E. & Hubbard, J. Anesthesia during and after exposure to microgravity. *Aviat. Space Environ. Med.* **75,** 571–80 (2004).	1				1	

15	Silverman, G. L. & McCartney, C. J. Regional Anesthesia for the Management of Limb Injuries in Space. *Aviat. Space Environ. Med.* **79,** 620–625 (2008).					● 1	
16	Robertson, D. *et al.* Clinical models of cardiovascular regulation after weightlessness. *Med. Sci. Sports Exerc.* **28,** S80-84 (1996).	● 1				● 1	
17	Johnston, S. L., Arenare, B. A. & Smart, K. T. Medical Evacuation and Vehicles for Transport. in *Principles of Clinical Medicine for Space Flight* (eds. M.D.,M.S, A. and P. M. R. B. & M.D, C. S. L. P.) 139–161 (Springer New York, 2008). doi:10.1007/978-0-387-68164-1_7	● 1					
18	Taddeo, T. A. & Armstrong, C. W. Spaceflight Medical Systems. in *Principles of Clinical Medicine for Space Flight* (eds. M.D.,M.S, A. and P. M. R. B. & M.D, C. S. L. P.) 69–100 (Springer New York, 2008).	● 1					
19	Campbell, M. R. *et al.* Endoscopic surgery in weightlessness: the investigation of basic principles for surgery in space. *Surg. Endosc.* **15,** 1413–1418 (2001).	● 1				● 1	
20	Otto, C. *et al.* Into thin air: extreme ultrasound on Mt Everest. *Wilderness Environ. Med.* **20,** 283–289 (2009).				● 1		
21	Otto, C. *et al.* The Martian chronicles: remotely guided diagnosis and treatment in the Arctic Circle. *Surg. Endosc.* **24,** 2170–2177 (2010).				● 1	● 1	
22	Sipes, W. E., Polk, J. D., Beven, G. & Shepanek, M. Behavioral Health and Performance. in *Space Physiology and Medicine* (eds. Nicogossian, A. E. et al.) 367–389 (Springer New York, 2016). doi:10.1007/978-1-4939-6652-3_14	● 1				● 1	
23	Nicogossian, A. E., Williams, D. R., Williams, R. S. & Schneider, V. S. Simulations and Analogs (Test-Beds). in *Space Physiology and Medicine* (eds. Nicogossian, A. E. et al.) 441–461 (Springer New York, 2016). doi:10.1007/978-1-4939-6652-3_17				● 1	● 1	
24	Simmons, S. C., Hamilton, D. R. & McDonald, P. V. Telemedicine. in *Principles of Clinical Medicine for Space Flight* (eds. M.D.,M.S, A. and P. M. R. B. & M.D, C. S. L. P.) 163–179 (Springer New York, 2008). doi:10.1007/978-0-387-68164-1_8	● 1				● 1	
25	World Bank. World Bank Country and Lending Groups. (2017). Accessed on 27/11/2017 https://datahelpdesk.worldbank.org/knowledgebase/articles/906519-world-bank-country-and-lending-groups http://www.webcitation.org/6vvcpSNBw		● 1				● 1
26	Bösenberg, A. T. Pediatric anesthesia in developing countries. *Curr. Opin. Anaesthesiol.* **20,** 204–210 (2007).		● 1				
27	Dubowitz, G., Detlefs, S. & McQueen, K. A. K. Global anesthesia workforce crisis: a preliminary survey revealing shortages contributing to undesirable outcomes and unsafe practices. *World J. Surg.* **34,** 438–444 (2010).		● 1				
28	Martin, J. *et al.* Survey of the capacity for essential surgery and anaesthesia services in Papua New Guinea. *BMJ Open* **5,** e009841 (2015).		● 1				

29	McQueen, K. *et al.* The Bare Minimum: The Reality of Global Anaesthesia and Patient Safety. *World J. Surg.* **39,** 2153–2160 (2015).		1				
30	Merchant, A. *et al.* Evaluating Progress in the Global Surgical Crisis: Contrasting Access to Emergency and Essential Surgery and Safe Anesthesia Around the World. *World J. Surg.* **39,** 2630–2635 (2015).		1				
31	WHO. *WHO Guidelines for Safe Surgery 2009: Safe Surgery Saves Lives* . (World Health Organization, 2009).		1				
32	*Essential Surgery: Disease Control Priorities, Third Edition (Volume 1)* . (The International Bank for Reconstruction and Development / The World Bank, 2015).		1				
33	Spiegel, D. A., Abdullah, F., Price, R. R., Gosselin, R. A. & Bickler, S. W. World Health Organization Global Initiative for Emergency and Essential Surgical Care: 2011 and beyond. *World J. Surg.* **37,** 1462–1469 (2013).		1				
34	Buckenmaier, C. C., Lee, E. H., Shields, C. H., Sampson, J. B. & Chiles, J. H. Regional anesthesia in austere environments. *Reg. Anesth. Pain Med.* **28,** 321–327 (2003).		1		1		
35	Keene, D. D. *et al.* Died of wounds: a mortality review. *J. R. Army Med. Corps* **162,** 355–360 (2016).			1			
36	Mahoney, P. F. *Combat Anesthesia: The First 24 Hours* . (Department of the Army, 2016).			1			
37	Mahoney, P. F. & McFarland, C. C. Field Anesthesia and Military Injury. in *Trauma Anesthesia* (Cambridge University Press, 2008).			1			
38	Mellor, A. J. Anaesthesia in austere environments. *J. R. Army Med. Corps* **151,** 272–276 (2005).			1	1		
39	National Association of Emergency Medical Technicians. Tactical Combat Casualty Care Guidelines for Medical Personnel. (2016).			1			
40	Imray, C. H. E., Grocott, M. P. W., Wilson, M. H., Hughes, A. & Auerbach, P. S. Extreme, expedition, and wilderness medicine. *The Lancet* **386,** 2520–2525 (2015).				1		1
41	Aaen-Larsen, B. Health care in the circumpolar world. Greenland. *Int. J. Circumpolar Health* **63 Suppl 2,** 49–53 (2004).				1		
42	Grant, I. C. Telemedicine in the British Antarctic survey. *Int. J. Circumpolar Health* **63,** 356–364 (2004).				1		
43	Otto, C. *et al.* Evaluation of tele-ultrasound as a tool in remote diagnosis and clinical management at the Amundsen-Scott South Pole Station and the McMurdo Research Station. *Telemed. J. E-Health Off. J. Am. Telemed. Assoc.* **19,** 186–191 (2013).				1		
44	Minard, C. G., de Carvalho, M. F. & Iyengar, M. S. Optimizing medical resources for spaceflight using the integrated medical model. *Aviat. Space Environ. Med.* **82,** 890–894 (2011).	1				1	
45	Fisher, A. D., Miles, E. A., Cap, A. P., Strandenes, G. & Kane, S. F. Tactical Damage Control Resuscitation. *Mil. Med.* **180,** 869–875 (2015).			1			
46	Grocott, M. & Johannson, L. Ketamine for emergency anaesthesia at very high altitude (4243 m above sea-level). *Anaesthesia* **62,** 959–962 (2007).				1		

47	Jochberger, S. *et al.* Anesthesia and its allied disciplines in the developing world: a nationwide survey of the Republic of Zambia. *Anesth. Analg.* **106,** 942–948, table of contents (2008).		● 1				
48	Wong, E. G. *et al.* Operative trauma in low-resource settings: The experience of Médecins Sans Frontières in environments of conflict, postconflict, and disaster. *Surgery* **157,** 850–856 (2015).		● 1				
49	Strube, P. D. & Perkins, A. D. Combat Anesthesia: A Case Report of a Gunshot Wound and New Trauma Protocols. *AANA J.* **83,** 247–253 (2015).			● 1			
50	WHO. Essential surgical care aide-memoire: surgical and emergency obstetrical care at first referral level. (2003).		● 1				
51	WHO. *Surgical Care at the District Hospital*. (World Health Organization, 2003).		● 1				
52	Clément, G. Psychological Issues of Spaceflight. in *Fundamentals of Space Medicine* 217–255 (Springer New York, 2011). doi:10.1007/978-1-4419-9905-4_6	● 1				● 1	
53	Shayler, D. *Disasters and Accidents in Manned Spaceflight*. (Springer, 2000).	● 1					
54	Crucian, B. *et al.* Immune System Dysregulation Occurs During Short Duration Spaceflight On Board the Space Shuttle. *J. Clin. Immunol.* **33,** 456–465 (2013).	● 1					
55	Mermel, L. A. Infection Prevention and Control During Prolonged Human Space Travel. *Clin. Infect. Dis.* **56,** 123–130 (2013).	● 1				● 1	
56	Smith, S. M. *et al.* Bone metabolism and renal stone risk during international space station missions. *Bone* (2015). doi:10.1016/j.bone.2015.10.002	● 1				● 1	
57	Komorowski, M., Fleming, S. & Hinkelbein, J. Anaesthesia in outer space: the ultimate ambulatory setting? *Curr. Opin. Anaesthesiol.* **29,** 649–654 (2016).	● 1				● 1	
58	Merry, A. F., Cooper, J. B., Soyannwo, O., Wilson, I. H. & Eichhorn, J. H. International Standards for a Safe Practice of Anesthesia 2010. *Can. J. Anaesth.* **57,** 1027–1034 (2010).		● 1				● 1
59	Pignaton, W. *et al.* Perioperative and Anesthesia-Related Mortality. *Medicine (Baltimore)* **95,** (2016).						● 1
60	Dobson, M. B. *Anaesthesia at the District Hospital*. (World Health Organization, 2001).		● 1				
61	Neuen, B. L. Access to Safe Anesthesia: A Global Perspective. *J. Glob. Health* (2014).		● 1				
62	Schnittger, T. Regional anaesthesia in developing countries. *Anaesthesia* **62,** 44–47 (2007).		● 1				
63	Wilhelm, T. J. *et al.* Anaesthesia for elective inguinal hernia repair in rural Ghana - appeal for local anaesthesia in resource-poor countries. *Trop. Doct.* **36,** 147–149 (2006).		● 1				
64	Ouro-Bang'na Maman, A. F., Agbétra, N., Egbohou, P., Sama, H. & Chobli, M. Morbidité–mortalité périopératoire dans un pays en développement : expérience du CHU de Lomé (Togo). *Ann. Fr. Anesth. Réanimation* **27,** 1030–1033 (2008).		● 1				
65	Eliassen, H. S. *et al.* Making whole blood available in austere medical environments: donor performance and safety. *Transfusion (Paris)* **56,** S166–S172 (2016).		● 1	● 1			

66	Ray, J. M. The Treatment of Maxillofacial Trauma in Austere Conditions. *Atlas Oral Maxillofac. Surg. Clin. North Am.* **21,** 9–14 (2013).		● 1				
67	Convertino, V. A. & Cooke, W. H. Evaluation of Cardiovascular Risks of Spaceflight Does Not Support the NASA Bioastronautics Critical Path Roadmap. *Aviat. Space Environ. Med.* **76,** 869–876 (2005).	● 1				● 1	
68	Epelman, S. & Hamilton, D. R. Medical Mitigation Strategies for Acute Radiation Exposure During Spaceflight. *Aviat. Space Environ. Med.* **77,** 130–139 (2006).	● 1				● 1	
69	Gillis, D. B. & Hamilton, D. R. Estimating outcomes of astronauts with myocardial infarction in exploration class space missions. *Aviat. Space Environ. Med.* **83,** 79–91 (2012).					● 1	
70	Hamilton, D. R. Cardiovascular Disorders. in *Principles of Clinical Medicine for Space Flight* (eds. Barratt, M. R. & Pool, S. L.) 317–359 (Springer New York, 2008).	● 1				● 1	
71	Gunga, H.-C., Ahlefeld, V. W. von, Coriolano, H.-J. A., Werner, A. & Hoffmann, U. The Cardiovascular System in Space. in *Cardiovascular System, Red Blood Cells, and Oxygen Transport in Microgravity* 11–34 (Springer International Publishing, 2016). doi:10.1007/978-3-319-33226-0_2	● 1				● 1	
72	Berwick, D. *et al.* *Creating and Sustaining an Expert Trauma Care Workforce*. (National Academies Press (US), 2016).			● 1			
73	Morey, T. E. & Rice, M. J. Anesthesia in an Austere Setting: Lessons Learned from the Haiti Relief Operation. *Anesthesiol. Clin.* **31,** 107–115 (2013).		● 1				
74	Trelles, M., Dominguez, L. & Stewart, B. T. Surgery in low-income countries during crisis: experience at Médecins Sans Frontières facilities in 20 countries between 2008 and 2014. *Trop. Med. Int. Health* **20,** 968–971 (2015).		● 1				
75	Dobson, M. B. DRAW-OVER ANAESTHESIA Part 2 - Practical Application. in *Update in Anaesthesia* (1993).		● 1				
76	Skelton, T. *et al.* Low-Cost Simulation to Teach Anesthetists' Non-Technical Skills in Rwanda. *Anesth. Analg.* **123,** 474–480 (2016).		● 1				
77	McQueen, K. *et al.* Anesthesia and Perioperative Care. in *Essential Surgery: Disease Control Priorities, Third Edition (Volume 1)* (eds. Debas, H. T. et al.) (The International Bank for Reconstruction and Development / The World Bank, 2015).		● 1				
78	Galvez, J. A. & Rehman, M. A. Telemedicine in anesthesia: an update. *Curr. Opin. Anaesthesiol.* **24,** 459–462 (2011).						● 1
79	Murray, A. W., Beaman, S. T., Kampik, C. W. & Quinlan, J. J. Simulation in the operating room. *Best Pract. Res. Clin. Anaesthesiol.* **29,** 41–50 (2015).						● 1
80	Ikeyama, T., Shimizu, N. & Ohta, K. Low-Cost and Ready-to-Go Remote-Facilitated Simulation-Based Learning. *Simul. Healthc.* **7,** 35 (2012).		● 1				● 1
81	Livingston, P. *et al.* Development of a simulation and skills centre in East Africa: a Rwandan-Canadian partnership. *Pan Afr. Med. J.* **17,** (2014).		● 1				

82	Sallie J. Weaver & Eduardo Salas. Training and Measurement at the Extremes: Developing and Sustaining Expert Team Performance in Isolated, Confined, Extreme Environments. *Proc. Hum. Factors Ergon. Soc. Annu. Meet.* **54,** 90–93 (2010).				● 1	● 1	
83	West, P., Gustin, N. & Raimer, B. *TELEMEDICINE LINK WITH SOUTH POLE ALLOWS REMOTE KNEE SURGERY* . (2002).				● 1		
84	Department of Defense. *Tactical Combat Casualty Care and Wound Treatment* . (Skyhorse Publishing, 2016).			● 1			
85	Komorowski, M., Neuhaus, C. & Hinkelbein, P. D. J. Emergency medicine in space. *Notf. Rettungsmedizin* **18,** 268–27[illegible] (2015).	● 1				● 1	
86	Saluja, I. S. *et al.* Survey of astronaut opinions on medical crewmembers for a mission to Mars. *Acta Astronaut* **63,** 586–593 (2008).					● 1	
87	Komorowski, M. & Fleming, S. Intubation after rapid sequence induction performed by non-medical personnel during space exploration missions: a simulation pilot study in a Mars analogue environment. *Extreme Physiol. Med.* **4,** 19 (2015).					● 1	
88	Beam, A. L. & Kohane, I. S. Translating Artificial Intelligence Into Clinical Care. *JAMA* **316,** 2368–2369 (2016).						● 1
89	Doarn, C. R., Williams, R. S., Nicogossian, A. E. & Polk, J. D. Training in Space Medicine. in *Space Physiology and Medicine* (eds. Nicogossian, A. E. et al.) 463–477 (Springer New York, 2016). doi:10.1007/978-1-4939-6652-3_18	● 1					
90	Jha, S. & Topol, E. J. Adapting to Artificial Intelligence: Radiologists and Pathologists as Information Specialists. *JAMA* **316,** 2353–2354 (2016).						● 1
91	Jones, P. M. Human Performance in Space. *Rev. Hum. Factors Ergon.* **6,** 172–197 (2010).	● 1				● 1	
92	Strangman, G. E., Sipes, W. & Beven, G. Human cognitive performance in spaceflight and analogue environments. *Aviat. Space Environ. Med.* **85,** 1033–1048 (2014).	● 1			● 1	● 1	
93	Walker, I. A. & Wilson, I. H. Anaesthesia in developing countries--a risk for patients. *Lancet Lond. Engl.* **371,** 968–969 (2008).		● 1				
94	Jawa, R. S., Zakrison, T. L., Richards, A. T., Young, D. H. & Heir, J. S. Facilitating safer surgery and anesthesia in a disaster zone. *Am. J. Surg.* **204,** 406–409 (2012).		● 1				
95	Vincent, J.-L. The Future of Critical Care Medicine: Integration and Personalization. *Crit. Care Med.* **44,** 386–389 (2016).						● 1
96	Ventola, C. L. Medical Applications for 3D Printing: Current and Projected Uses. *Pharm. Ther.* **39,** 704–711 (2014).					● 1	● 1
97	Mehta, P. & Bhayani, D. Impact of space environment on stability of medicines: Challenges and prospects. *J. Pharm. Biomed. Anal.* **136,** 111–119 (2017).					● 1	
98	Spinella, P. C. Warm fresh whole blood transfusion for severe hemorrhage: U.S. military and potential civilian applications. *Crit. Care Med.* **36,** S340-345 (2008).			● 1			
99	Mahmood, F. *et al.* Perioperative Ultrasound Training in Anesthesiology: A Call to Action. *Anesth. Analg.* **122,** 1794–1804 (2016).						● 1

100	Talbot, M., Harvey, E. J., Reindl, R., Martineau, P. & Schneider, P. Ultrasound-assisted external fixation: a technique for austere environments. *J. R. Army Med. Corps* **162,** 456–459 (2016).			1			
101	Nguyen, B.-V. *et al.* Determination of the learning curve for ultrasound-guided jugular central venous catheter placement. *Intensive Care Med.* **40,** 66–73 (2014).						1
102	Bye, S. M. & Manankov, A. Telemedicine in practice in Arkhangelsk region, Russia: from a blank page to routine operation. *Int. J. Circumpolar Health* **66,** 335–350 (2007).				1		
103	Hemmerling, T. M. *et al.* Evaluation of a novel closed-loop total intravenous anaesthesia drug delivery system: a randomized controlled trial. *Br. J. Anaesth.* **110,** 1031–1039 (2013).						1
104	Baker, E. S., Barratt, M. R. & Wear, M. L. Human Response to Spaceflight. in *Principles of clinical medicine for space flight* (eds. Barratt, M. R. & Pool, S. L.) 27–58 (Springer, 2008).	1				1	
105	Lee, S. M. C., Feiveson, A. H., Stein, S., Stenger, M. B. & Platts, S. H. Orthostatic Intolerance After ISS and Space Shuttle Missions. *Aerosp. Med. Hum. Perform.* **86,** A54-67 (2015).	1				1	
106	Hargens, A. R. & Richardson, S. Cardiovascular adaptations, fluid shifts, and countermeasures related to space flight. *Respir. Physiol. Neurobiol.* **169 Suppl 1,** S30-33 (2009).	1				1	
107	Norfleet, W. Anesthetic Concerns of Spaceflight. *Anesthesiology* **98,** 1219 (2000).	1				1	
108	Modi, P. *et al.* Accuracy of Inferior Vena Cava Ultrasound for Predicting Dehydration in Children with Acute Diarrhea in Resource-Limited Settings. *PLoS ONE* **11,** (2016).		1				
109	Chisholm, C. B. *et al.* Focused Cardiac Ultrasound Training: How Much Is Enough? *J. Emerg. Med.* **44,** 818–822 (2013).						1
110	Buis, M. L., Maissan, I. M., Hoeks, S. E., Klimek, M. & Stolker, R. J. Defining the learning curve for endotracheal intubation using direct laryngoscopy: A systematic review. *Resuscitation* **99,** 63–71 (2016).						1
111	Lascarrou, J. B. *et al.* Video Laryngoscopy vs Direct Laryngoscopy on Successful First-Pass Orotracheal Intubation Among ICU Patients: A Randomized Clinical Trial. *JAMA* **317,** 483–493 (2017).						1
112	Lewis, S. R., Butler, A. R., Parker, J., Cook, T. M. & Smith, A. F. Videolaryngoscopy versus direct laryngoscopy for adult patients requiring tracheal intubation. *Cochrane Database Syst. Rev.* **11,** CD011136 (2016).						1
113	Aguirre Ospina, O. D., Ríos Medina, Á. M., Calderón Marulanda, M. & Gómez Buitrago, L. M. Cumulative Sum learning curves (CUSUM) in basic anaesthesia procedures. *Colomb. J. Anesthesiol.* **42,** 142–153 (2014).						1
114	Nouruzi-Sedeh, P., Schumann, M. & Groeben, H. Laryngoscopy via Macintosh blade versus GlideScope: success rate and time for endotracheal intubation in untrained medical personnel. *Anesthesiology* **110,** 32–37 (2009).						1
115	Rudraraju, P. & Eisen, L. A. Confirmation of endotracheal tube position: a narrative review. *J. Intensive Care Med.* **24,** 283–292 (2009).						1

116	Komatsu, R. *et al.* Learning Curves for Bag-and-mask Ventilation and Orotracheal IntubationAn Application of the Cumulative Sum Method. *Anesthesiol. J. Am. Soc. Anesthesiol.* **112,** 1525–1531 (2010).						● 1
117	Chesters, A. & Webb, T. Ketamine for procedural sedation by a doctor-paramedic prehospital care team: a 4-year description of practice. *Eur. J. Emerg. Med. Off. J. Eur. Soc. Emerg. Med.* **22,** 401–406 (2015).				● 1		
118	Bishop, R. A., Litch, J. A. & Stanton, J. M. Ketamine anesthesia at high altitude. *High Alt. Med. Biol.* **1,** 111–114 (2000).				● 1		
119	Mulvey, J. M., Qadri, A. A. & Maqsood, M. A. Earthquake injuries and the use of ketamine for surgical procedures: the Kashmir experience. *Anaesth. Intensive Care* **34,** 489–494 (2006).		● 1				
120	Russell, K. W. *et al.* Wilderness Medical Society Practice Guidelines for the Treatment of Acute Pain in Remote Environments. *Wilderness Environ. Med.* **25,** 41–49 (2014).				● 1		
121	Burgess, C. & Dubbs, C. Cosmos/Bion: The age of the biosatellites. in *Animals in Space* 277–305 (Springer New York, 2007). doi:10.1007/978-0-387-49678-8_10	● 1					
122	Groemer, G. E. *et al.* The Feasibility of Laryngoscope-Guided Tracheal Intubation in Microgravity During Parabolic Flight: A Comparison of Two Techniques. *Anesth. Analg.* **101,** 1533–1535 (2005).	● 1				● 1	
123	Keller, C. *et al.* Airway management during spaceflight: A comparison of four airway devices in simulated microgravity. *Anesthesiology* **92,** 1237–1241 (2000).	● 1				● 1	
124	Rabitsch, W. *et al.* Airway management with endotracheal tube versus Combitube during parabolic flights. *Anesthesiology* **105,** 696–702 (2006).	● 1				● 1	
125	Adesunkanmi, A. R. Where there is no anaesthetist: a study of 282 consecutive patients using intravenous, spinal and local infiltration anaesthetic techniques. *Trop. Doct.* **27,** 79–82 (1997).				● 1		
126	Aluisio, A. R., Teicher, C., Wiskel, T., Guy, A. & Levine, A. Focused Training for Humanitarian Responders in Regional Anesthesia Techniques for a Planned Randomized Controlled Trial in a Disaster Setting. *PLOS Curr. Disasters* (2016). doi:10.1371/currents.dis.e75f9f9d977ac8adededb381e3948a04		● 1				
127	Newton, M. & Bird, P. Impact of Parallel Anesthesia and Surgical Provider Training in Sub-Saharan Africa: A Model for a Resource-poor Setting. *World J. Surg.* **34,** 445–452 (2010).		● 1				
128	Kessler, J., Wegener, J. T., Hollmann, M. W. & Stevens, M. F. Teaching concepts in ultrasound-guided regional anesthesia. *Curr. Opin. Anaesthesiol.* **29,** 608–613 (2016).						● 1
129	Luyet, C. *et al.* Different Learning Curves for Axillary Brachial Plexus Block: Ultrasound Guidance versus Nerve Stimulation. *Anesthesiol. Res. Pract.* **2010,** (2010).						● 1
130	Hunter, J. G. Managing pain on the battlefield: an introduction to continuous peripheral nerve blocks. *J. R. Army Med. Corps* **156,** 230–232 (2010).			● 1			

131	Chou, R. *et al.* Management of Postoperative Pain: A Clinical Practice Guideline From the American Pain Society, the American Society of Regional Anesthesia and Pain Medicine, and the American Society of Anesthesiologists' Committee on Regional Anesthesia, Executive Committee, and Administrative Council. *J. Pain* **17,** 131–157 (2016).						1
132	*Artificial Gravity* . (Springer, 2007).					1	
133	Barash, P. G. *et al.* *Clinical Anesthesia, 8e: Print + Ebook with Multimedia* . (LWW, 2017).						1
134	Miller, R. D. *et al.* *Miller's Anesthesia, 2-Volume Set, 8e* . (Saunders, 2014).						1

Key:
LEO: Low Earth Orbit
LMIC: Low and Middle Income Country
ICE: Isolated and Confined Environment
SEM: Space Exploration Mission
